# β-Glucan as a Techno-Functional Ingredient in Dairy and Milk-Based Products—A Review

**DOI:** 10.3390/molecules27196313

**Published:** 2022-09-24

**Authors:** Artur Mykhalevych, Galyna Polishchuk, Khaled Nassar, Tetiana Osmak, Magdalena Buniowska-Olejnik

**Affiliations:** 1 Department of Milk and Dairy Products Technology, National University of Food Technologies, Volodymyrska St. 68, 01033 Kyiv, Ukraine; 2 Faculty of Agriculture, Damanhour University, Damanhour 22516, Egypt; 3 Department of Dairy Technology, Institute of Food Technology and Nutrition, University of Rzeszow, Ćwiklinskiej 2D St., 35601 Rzeszow, Poland

**Keywords:** polysaccharides, microalgae, mushrooms, yeast, bioactive substances, ice cream, cheese, fermented milk products, milk drinks, β-glucan

## Abstract

The article systematizes information about the sources of β-glucan, its technological functions and practical aspects of its use in dairy and milk-based products. According to the analysis of scientific information, the main characteristics of β-glucan classifications were considered: the source of origin, chemical structure, and methods of obtention. It has been established that the most popular in the food technology of dairy products are β-glucans from oat and barley cereal, which exhibit pronounced technological functions in the composition of dairy products (gel formation, high moisture-binding capacity, increased yield of finished products, formation of texture, and original sensory indicators). The expediency of using β-glucan from yeast and mushrooms as a source of biologically active substances that ensure the functional orientation of the finished product has been revealed. For the first time, information on the use of β-glucan of various origins in the most common groups of dairy and milk-based products has been systematized. The analytical review has scientific and practical significance for scientists and specialists in the field of food production, in particular dairy products of increased nutritional value.

## 1. Introduction

β-glucan is a polysaccharide found naturally in the cell walls of cereals, yeasts, seaweeds, bacteria, and fungi. The physicochemical, functional, and technological properties of β-glucan are extremely different, depending on the source of origin [1]. This polysaccharide is used in the therapeutic, cosmetic, fitness, and professional sports fields [2,3]. Interest in β-glucan has arisen because it is a powerful immunostimulant, prebiotic, and dietary fiber [4,5]. Interest in the use of β-glucan in the food industry is associated not only with its positive impact on the health of consumers but also with its functional and technological properties, which significantly improve the consumer characteristics of food products.

The use of oat β-glucan in the food industry became possible when the EFSA confirmed in 2010 that the daily consumption of oat β-glucan in the amount of 3 g can reduce the risk of coronary disease and have a positive effect on the cardiovascular system, provided that a diet with low saturated fat content is followed [6]. β-glucan made from yeast has been recognized as a novel ingredient and authorized for release since 2011 [7].

β-glucan, as polysaccharide, built only from glucose monomers is versatile and multifunctional, but its characteristics, strongly dependent on botanical origin, is still unsufficiently investigated, especially considering the mutual interactions of different recipe components. β-glucan is a very promising techno-functional ingredient for food industry, but the data characterizing its properties are too dispersed in the scientific literature and need to be organized to be useful for food professionals. Special attention is paid to research on the rheological properties of β-glucan because of its ability to form gels and increase the viscosity of products during technological processing due to its high structuring ability, which can be useful when researching low-fat food systems [8]. The regularities of the effect of molecular weight, pH, temperature, shear rate, and the duration of the breaking spatial bonds process on the structuring ability and thixotropy of food systems with β-glucan remain debatable issues and require further study [9].

Triton Market Research concluded that the β-glucan market was assessed to be worth 340.63 million USD in 2018 and is predicted to generate a revenue of approximately 656.65 million USD by 2027, which includes the dairy industry as one of the branches that needs β-glucan as a technological ingredient.

In market conditions with a growing demand for healthy food and low-calorie products (most often due to a decrease in the mass fraction of fat), the use of ingredients capable of imitating the organoleptic properties of high-fat analogs, as well as improving the physicochemical properties of dairy and milk-based products, is relevant [10]. Fat significantly affects the quality indicators of food products, so reducing its content or excluding it from the composition of food products inevitably leads to a deterioration in their quality [11]. 

Scientists have repeatedly proven that polysaccharides, in particular β-glucan, are technologically effective ingredients in dairy and milk-based products, which allows them to be used both individually and in combination with proteins of various origins, pectins, and other substances [12,13]. Different methods of processing β-glucan, intermediate raw materials with it, or products in the recipe in which it is contained can give it new functional and technological properties. However, the number of studies devoted to the understanding of the β-glucan specifics used in dairy product technologies is quite small, which confirms the need for further development in this scientific direction [14].

Because of this, the organization of scientific knowledge about the chemical structure and properties of β-glucan from different sources in dairy and milk-based products will help advance these technologies in line with current food trends.

Considering the fact that the use of β-glucan in the food industry, and in particular the dairy, is a promising direction, and the existing information in this area is not systematized, the analysis of all existing recommendations, methods, and advantages of using this polysaccharide of various origins in the composition of certain types of dairy products is relevant. The results of this analytical review can have scientific and practical significance in the field of food science and the industrial production of dairy products.

## 2. The Characteristics of β-Glucan of Various Origins 

### 2.1. Sources of β-Glucan

The amount of β-glucan that can be obtained from natural sources depends on the method and degree of purification, technology, and growing conditions (for grain crops), as well as on the source of β-glucan itself, which determines its chemical structure and functional and technological properties. The information on the content of β-glucan in the main raw materials, systematized by the authors based on an analysis of research works, is given in Table 1. 

One of the most famous sources of β-glucan is cereal crops: oats, barley, rye, wheat, etc., which explains the largest number of research papers with this raw material [24]. However, this information is not enough to answer all of the questions that scientists and food technologists have about how β-glucan can be used in different food systems. The possibility of using other types of β-glucan in the technology of dairy products, in particular their impact on organoleptic indicators, as well as bioavailability and the impact on the human body, are still an important question.

Without special processing methods, up to 6% of β-glucan can be obtained from grain crops, depending on the type of plant (Table 1). Increasing the yield of β-glucan is possible with the use of genotyping technology, which has found application for such grain crops as oats, barley, and wheat and provides raw materials with the average content of this polysaccharide at a level of 6–9% for oats and barley and 1–1.5% for wheat [27,28,29]. Separate patented methods of genotyping can allow increasing the content of β-glucan even more. Thus, Stephen Alan Jobling and his colleagues invented a method of obtaining wheat grain with a β-glucan content of more than 3% [44], which is based on recombinant DNA technology.

### 2.2. Chemical Structure

The chemical structure of β-glucan in cereals is an unbranched polysaccharide formed from glycopyranose residues connected by β-(1→4) bonds and isolated β-(1→3) bonds [45]. Isolated β-(1→4) bonds are not found in the structure of grain β-glucans. Most β-(1→4) linkages are arranged in groups of two or three [46]. The main structural fragment is cepicellotriose and cellotetraose residues connected by single β-(1→3) bonds [47]. The main chain of β-glucan thus resembles the structure of cellulose but has a bend in the position of the β-(1→3) linkage, as a result of which the strong hydrogen bonds that are present in cellulose are destroyed [48]. This explains the solubility of β-glucan from cereals in water.

The extraction of β-glucan from bacterial cell walls is one technological innovation that lacks sufficient scientific data [49]. Thus, Gemilang Lara Utama et al. [50] state that the β-(1→3)-glucan component in the cell walls of yeasts and bacteria such as Xanthomonas campertris and Bacillus sp. is much smaller than that of the fungus, as a result of which the yield of β-glucan from such a source is lower. However, such representatives of bacteria are usually able to produce structuring homo- or heteropolysaccharides capable of forming gels in food systems [51].

Bacterial β-glucan have a straight and unbranched β-(1→3)-D-glucan structure, while β-glucan from seaweed can contain a straight chain of β-(1→3) residues or a straight chain of β-(1→6)-linked glucosyl side branches [52,53]. 

It should be noted that the content of β-glucan in some species of microalgae, such as Euglena, can reach 90%, which usually depends on the quality of the raw material and the method of extraction [54]. Using excess irradiation of Euglena microalgae [43] or culturing cells in a certain growth medium and special conditions [55], it is possible to obtain the maximum yield of β-glucan at a level of 90%, while extraction by traditional methods provides a yield of 20–70%, depending on which morphological part of the microalgae was used.

The content of β-glucan in yeast, such as Saccharomyces cerevisiae, is higher than in cereal crops and is 55–65% [31,33]. Their chemical structure is represented by a complex of linear β-(1→3) chains with residual straight chains connected to them by long branches connected through β-(1→6) bonds [52].

Edible mushrooms can also be a source of β-glucan. However, most mushrooms do not have a high content of this polysaccharide (up to 1%); although some of them, such as Gyrophora esculenta, contain more than 40% β-glucan. 

The rest of the fungi vary in β-glucan content, from those containing it at the level of wheat to those containing up to 20% or more. They are mostly looked at as a source of β-glucan in the biomedical and pharmaceutical fields. Krzysztof Sobieralski et al. [56] proved that mushrooms are extremely promising for obtaining a biologically active form of β-glucan, which can be a component in the formulation of functional food products, in particular dairy products [57]. It can be explained by the fact that these compounds have another structure, water solubility, and molecular mass, which determine their medicinal properties. β-glucans made from mushroms show a very wide range of health-supporting activity.

β-glucan of fungal origin contain β-(1→6)-linked chains extending from a β-(1→3) backbone. It should be noted that the basic structure of β-glucans also depends on the fungal source: fungal β-glucans have short β-(1→6)-linked chains, while yeast has β-(1→6) side chains with additional β-(1→3) chains [45,58]. These differences affect the properties of β-glucan as an immunoprotector and also further determine its ability to suppress the development of pathogenic microflora [57]. However, β-glucans made from mushrooms are not registered for medical use, so studies that will confirm the possibility of their further use are ongoing.

**Figure 1:**
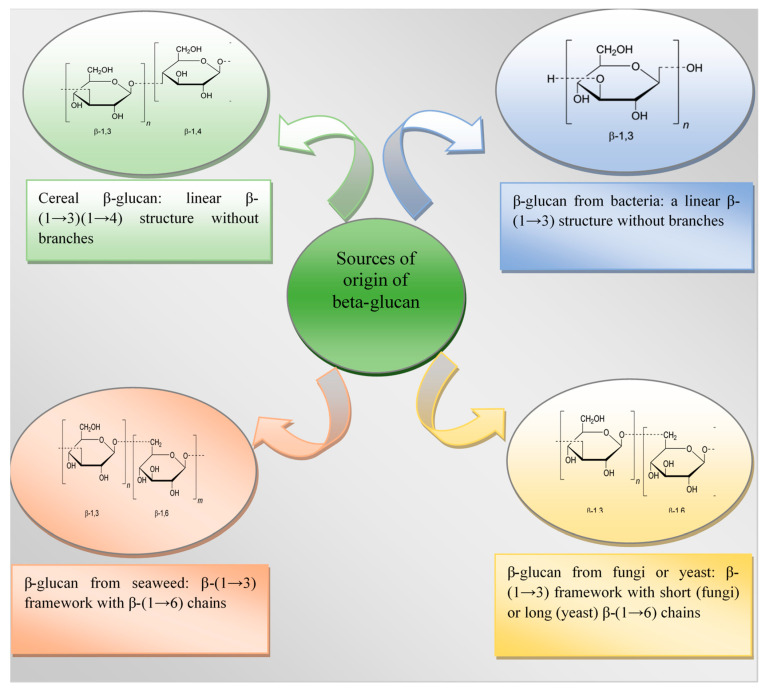
Chemical structure of β-glucan depending on the source of origin [59,60].

The chemical structure of β-glucan of various types is illustrated in Figure 1.

### 2.3. Techno-Functional Properties

According to the scientific database PubMed, the number of scientific works on the search term “β-glucan” is 18,039 articles (from 1953 to 2021, inclusive), of which the results of 4283 works were published in the last 5 years, which indicates that it is generally understudied, because interest in β-glucan increased only during the last 2–3 years.

**Figure 2:**
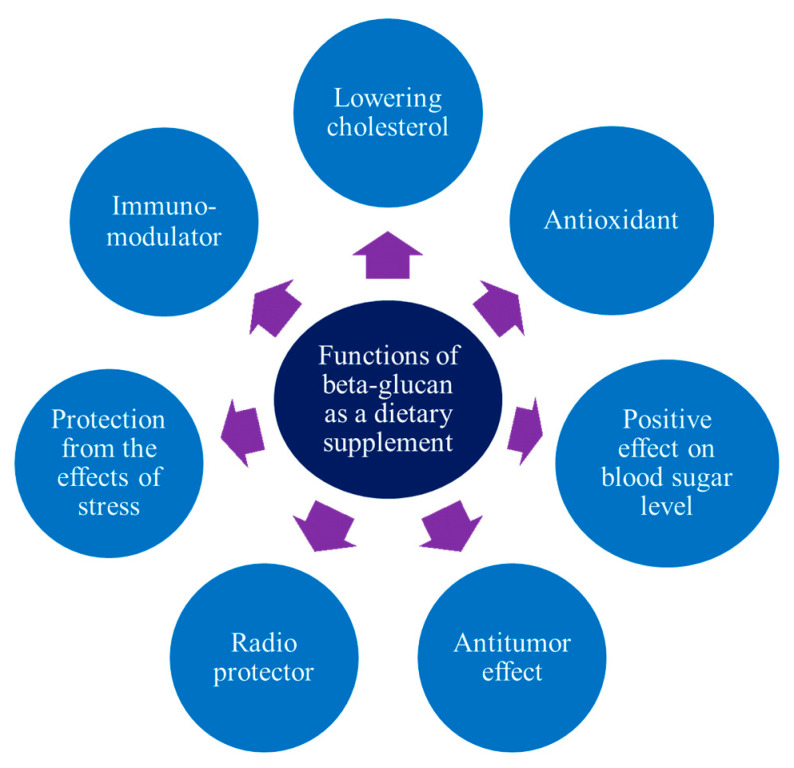
Functional properties of the β-glucan of various types.

β-glucan is currently not widely used in the food industry [61], which is due to the insufficient awareness of manufacturers regarding its functional and technological properties. Most experts associate β-glucan with a biologically active additive that is used for therapeutic purposes or as part of various diets for people with diabetes, obesity, and cardiovascular diseases. This makes sense because there have been a lot more clinical studies on the chemical makeup of β-glucan and how it affects the way the human body works (Figure 2) than on how it is used in food technology [62].

The composition of foods such as yeast, mushrooms, seaweed, and cereals (oat, barley, rye) includes complex carbohydrates, polysaccharides called β-glucans [60]. They were discovered and studied in the middle of the 20th century almost simultaneously in the USA and Japan. The use of medicinal mushrooms in Asian medicine has a long tradition. In a detailed study of the biological effects of these fungi, it was found that the presence of β-glucans in them is an important factor in nonspecific immunomodulation [57,58].

Most research on the use of β-glucan, as a technological ingredient, has been conducted in the field of bread and bakery products [63]. As for dairy products, the most common application for their use are in yogurts and yogurt drinks, fermented and non-fermented sports drinks, white-brined cheeses, and ice cream. 

**Figure 3:**
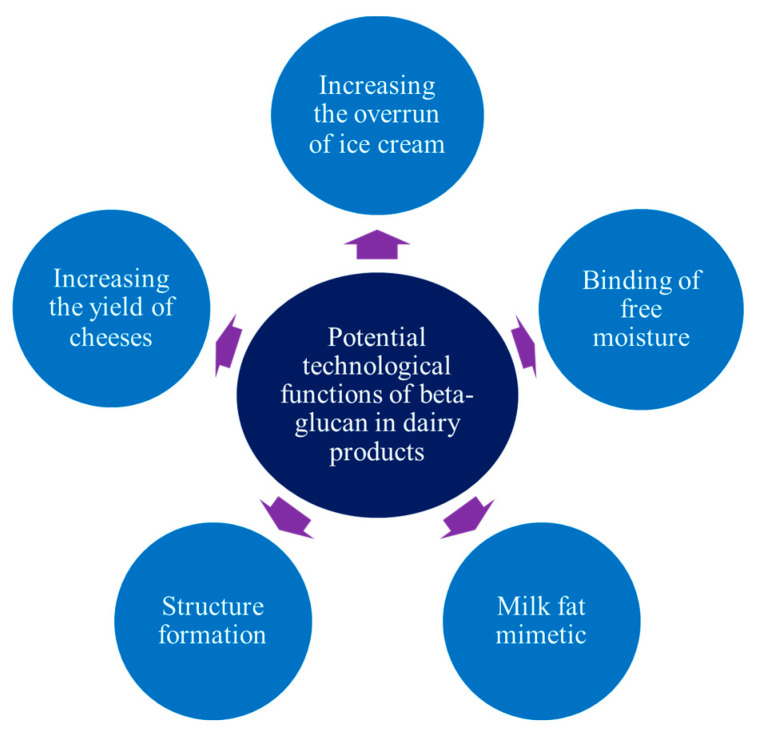
Technological properties of the β-glucan of different types.

This mostly concerns the use of oat and barley β-glucan [4], while β-glucan of yeast, bacteria, and fungi is little-researched, for example, in ice cream technology. At the same time, this ingredient opens a new field for scientific developments, considering its potential technological properties in the composition of many dairy and milk-based products, such as formation of a texture, original sensory properties, and others (Figure 3).

*2.4 Recommended Dosage of β-glucan for Dairy Products*

The authors systematized the recommended dosage ranges of β-glucan of various origins for dairy and milk-based products based on the analysis of scientific developments using this additive (Table 2, Table 3 and Table 4). In the next parts of this article, we will talk in more depth about the techno-functional properties of β-glucan in dairy products at the following doses.

It should be noted that the given data (Table 2, Table 3 and Table 4) are generalized ranges of β-glucan content in dairy products. When planning an experiment using this additive, it is necessary to take into account not only its origin and chemical structure but also the recipe composition of the product and the compatibility of other components with β-glucan [89], as well as the purpose of its use—to achieve a certain technological effect and/or provide the product with a functional orientation.

## 3. The Use of β-Glucan in the Technology of Dairy Drinks and Fermented Milk Products

### 3.1. Non-Fermented Milk Drinks

The production of chocolate milk is usually associated with the use of carrageenan as a stabilizer capable of reducing the gravitational settling of cocoa powder particles. Bandana Chatterjee and Tinkal Patel [64] proved that the use of oat β-glucan in the amount of 3% in combination with carrageenan increases the viscosity, has a favorable effect on the sensory indicators of chocolate milk, and also enriches the product with dietary fiber, which allows positioning such a drink as functional. 

In the technology of milk with fillers—banana, chocolate, vanilla, strawberry—it is possible to use barley β-glucan, since it has a chemical structure similar to oat β-glucan. In addition, it is possible to completely replace carrageenan with β-glucan as it is an effective structure-former, which has been proven in experimental work with meat emulsions [90].

Oat β-glucan can act as a substitute for guar gum. Eva Vasquez-Orejarena et al. [65] developed a composition for a high-protein milk drink in which oat flour was used as a stabilizer and a source of β-glucan. It was established that the combination of milk protein isolate in the amount of 2.5% and 1.9% of oat flour (0.75 g oat β-glucan per 1 serving) provided high-suspension stability for the drink (> 80%) and viscosity inherent in liquid drinks (< 50 mPa·s) with stabilizers [65]. An increase in the amount of oat flour led to a significant increase in the viscosity of protein drinks (from 51 to 100 mPa·s), which negatively affects their taste perception. Researchers have repeatedly drawn the attention of manufacturers to the fact that the design of recipes for new drinks, in particular milk drinks, with dietary fiber, such as β-glucan, requires a systematic approach to determining the rational dose of the additive [91]. The functional properties of dietary fiber and marketing promotion make it a popular product, which immediately affects the desire of manufacturers to include it in the composition of products and position them as healthy or enriched [92]. However, exceeding the rational dose of β-glucan in cereals leads to the formation of a sandy consistency of the product, an aftertaste of oatmeal, or dryness in the mouth [93].

The combination of a protein ingredient with cereal β-glucan has been studied in the composition of a milk-based functional drink [66]. Orange-juice-based drink samples contained 0.5% barley β-glucan and from 0 to 1.5% whey protein isolate. Due to the protein and β-glucan content, the drink becomes more structured, less acidic, and somewhat loses its orange taste. This is partly explained by the ability of β-glucan to neutralize the sour taste, which is confirmed by the results of the sensory evaluation of pasta with β-glucan [94], but the mechanism of this action requires additional research. β-glucan prevents the delamination of food systems due to its structuring ability, which is confirmed by the stability of the physicochemical parameters during storage. Similar data are given by Marika Lyly et al. [95], based on the results of the study of the β-glucan effects with different degrees of purification on the viscosity of food systems classified as beverages. Thus, preparations with a cereal β-glucan content in the range of 13.4–13.7% for an excess of 0.5% lead to the excessive viscosity of food systems, which makes them unsuitable for positioning as liquid drinks, while preparations with a higher degree of purification (21.9–34.2%) allow their use in amounts up to 2%, provided that traditional structure-formers, such as starch, carrageenan, and guar gum are excluded.

### 3.2. Fermented Dairy Products

The use of oat β-glucan in fermented milk drinks (kefir, yogurts, rhazhenka, acidophilic milk, etc.) is promising due to the fact that it allows the improvement of the physicochemical parameters of the product, in particular, to increase viscosity, reduce acidity, prevent consistency defects (the separation of free water and the delamination of the product) [69], as well as provide original taste properties. Thus, it was indicated that 0.6% of oat β-glucan in the composition of kefir, yogurt, and fermented milk drinks based on buttermilk and skimmed milk significantly increases the viscosity, especially in yogurt [68]. Furthermore, it was found that kefir drink and fermented milk had the best taste properties, while yogurt acquired a pronounced extraneous aftertaste of rice porridge. A dose of 0.6% oat β-glucan excessively increases the viscosity of the drink, which makes it impossible for the fermentation process to take place effectively. Xiaoqing Qu et al. [69] claim that a dose of oat β-glucan at the level of 0.3% somewhat changes the chemical structure of three-dimensional mesh structure of yogurt due to the fact that oat β-glucan slows down the interaction with casein, which shortens the fermentation process by 16 min and increases the taste properties of the product. At the same time, a study by other scientists indicates that oat β-glucan in the amount of 1.4% does not provide proper structuring of yogurt and leads to a liquid consistency of the product [94], which can be explained by a specific combination of lactic acid cultures [84], a reduced fermentation temperature (36 °C), and an increased dose of polysaccharide, which was due to the desire to position the product as functional in terms of its dietary fiber content, but without a scientific explanation of the product formulation, it is impossible. Thus, the addition of oat β-glucan to milk before fermentation slows protein aggregation due to phase separation between milk proteins and β-glucan, which leads to a decrease in gelation [96]. Therefore, when using cereal β-glucan with relatively high content, it is advisable to additionally use probiotic strains of microorganisms that will ensure proper gel formation. Furthermore, the increased amount of oat β-glucan has a positive effect on the growth and development of microorganisms such as *L. Paracasei* [94].

The influence of cereal β-glucan on the development and vital activity of probiotic organisms in the composition of fermented milk and milk-based drinks was also noted by other scientists. María Isabel Chávez de la Vega et al. [97] reported that oat β-glucan affects the proteolytic activity of *Lb. Rhamnosus* GG during milk fermentation. In order to achieve the maximum impact on the development of *Lb. Rhamnosus* GG, β-glucan content should be 22.46 g per 1 liter of milk [97]. Poorva Sharma et al. [98] established that the number of bacterial cells of *Lactobacillus acidophilus* and *Lactobacillus bulgaricus* during fermentation of mixtures with whey protein concentrate (70%) and oat β-glucan significantly increases during the first 10 hours of the process. Similar are the conclusions regarding the significant influence of oat β-glucan on the vital activity of *Lactobacillus plantarum* B28 in the composition of a probiotic drink made from oats [99].

Most scientists confirm that one of the main advantages of using cereal β-glucans as part of fermented milk products is their effect on syneresis [31], which is especially relevant in the production of low-fat or skimmed fermented milk products. The difference in the degree of influence of oat or barley β-glucan on the viscosity of yogurts [100] can be explained by the composition of their formulations. It was established that, in the presence of starch, the hydrophobicity of the hydrogen bonds of amylose and β-glucan occurs, which leads to the destabilization of the spatial network and, as a result, the liquid consistency of the drink [101]. This shows once again that the ingredients in products with β-glucan need to be backed up by science.

The use of β-glucan from baker’s yeast in the technology of milk drinks allows obtaining healthy products. Eunice Mah et al. [67] developed a milk drink with 0.1% β-glucan from dispersed yeast, which was included in the diet of marathon runners. It has been established that the consumption of such a drink during the 91^st^ day reduces the symptoms of a cold a few days after intense exertion, which allows reducing training gaps after a marathon and recovering strength sooner. Eunice Mah et al. [67] investigated soluble and insoluble β-glucan from Wellmune® brand yeast at 0.1% in a milk drink that improved symptoms in marathon runners. The results of the conducted research confirm that not all β-glucan is able to show immunomodulating properties. In particular, this is more characteristic of β-glucans from fungi and yeast [102]. The yeast preparation, Wellmune®, is widely used in various fields of the food industry and pharmaceuticals [103,104], but it is understudied in the technology of milk drinks, in particular for therapeutic and medicinal purposes.

The use of brewer’s yeast as a source of β-glucan is rational not only from the point of view of improving the physicochemical parameters of the product but also from the point of view of implementing the principles of sustainable development of brewing enterprises [105]. Brewer’s yeast is a by-product of beer production, the volume of which is extremely large, which can have a negative impact on the environment [106,107]. 

β-glucan from brewer’s yeast was studied in the range of 0 to 2% in the composition of skimmed milk yogurt [74]. It was established that its dose at a level of 1.5% improves the rheological properties, in particular, it allows to obtain the viscosity value and consistency characteristic of yogurt with high-fat content. The possibility of using β-glucan from brewer’s yeast as a milk fat replacer was investigated by Anna Piotrowska et al. [70] in the formulation of yogurt with 3% fat. Among the range of β-glucan (0.15–0.9%) chosen for the study, the best dose was 0.3%, which provided a rich milky taste, viscous consistency, and milky smell. Such a result of the sensory evaluation was possible only because the yogurt was not low-fat.

In another study, it was found that yeast β-glucan in low-fat yogurt is an effective thickener and reduces fermentation time by 25%, which can be explained by the property of β-glucan from brewer’s yeast to form small-sized clusters in the yogurt matrix [108]. Increasing its dose to 0.8% helps to improve the sensory properties of the product but does not significantly affect the physicochemical properties, such as syneresis, titrated acidity, and viscosity.

The source of β-glucan can be yeast sediment obtained during the production of wines [109], for example, Viorica wine (Moldova), the amount of which was determined by the laboratory method and was (28.17 ± 0.32)% [75]. Its addition to low-fat yogurt in the amount of 0.2–0.5% provides a reduction in the fermentation process by 1 hour (total time: 5 hours), which is due to the gel-forming ability of β-glucan [110]. However, this amount does not significantly affect the rheological parameters and is not able to act as a substitute for milk fat, which was also reported in other experiments [108].

The extraction of β-glucan from edible mushrooms and its use in the food industry is a promising direction in the development of technologies for healthy food products containing biologically active substances, vitamins, and mineral complexes in their chemical composition [111].

Bernadetta Hozová et al. [71] investigated the quality of fruit yogurts with β-glucan hydrogels from *Pleurotus ostreatus* pleura and *Lentinus edodes* lentinan. The experiment demonstrated the ability of these β-glucan hydrogels in the amount of 0.5 mL per 150 mL of yogurt to suppress the development of coliform bacteria, yeast, and mold, but no positive effect on the vital activity of sourdough cultures was noted. Such data correlates with the scientific statements of other scientists regarding the ability of β-glucans from edible mushrooms to inhibit the growth of pathogenic microorganisms and exert an anti-allergic, antioxidant effect [112].

β-glucan extraction from *Pleurotus citrinopileatus* mushrooms was investigated in low-fat yogurt technology [76]. Scientists have developed pasty β-glucan and used it in yogurts in the amount of 0.3–0.5%. According to the results of the experiment, a rational dose of β-glucan of 0.3% was established, the excess of which reduced the viscosity and sensory properties.

The use of β-glucan from *Ganoderma lucidum* mushrooms as part of therapeutic yogurt allows for protecting the body of children aged 3–5 years from infectious diseases [72], and its consumption in the amount of 1% with *Plukenetia volubilis* seeds in yogurt based on skimmed milk powder significantly improves organoleptic properties, even though there was some reduction in rheological characteristics [113]. This dose allows to simulate the presence of fat in yogurt and brings it closer to the control sample in terms of taste perception.

In general, the use of β-glucan from edible mushrooms in dairy beverage technologies is quite limited due to the lack of information on its properties in food systems based on dairy raw materials, as well as the complex production technology (paste- or gel-like form).

The use of β-glucan from microalgae in food technology is also poorly researched. One such representative is *Euglena gracilis*, which contains a significant amount of β-glucan (> 50%) [114]. The use of this microalgae became possible when the NDA in 2020 recognized the safety of dried whole cells of *Euglena gracilis* as an innovative ingredient, which confirmed the regulation of the European Commission [115]. The permissible levels of use of *Euglena gracilis* in fermented milk drinks are as follows: yogurt–no more than 150 mg/100 g, yogurt drinks–no more than 93.75 mg/100 g [73].

The effect of biologically active substances of *Euglena gracilis* on the vital activity of lactobacilli is known, but scientists have not yet determined what role β-glucan plays in this process. Junjie Dai et al. [116] believe that β-glucan support for the growth of bacteria such as *Lactobacillus acidophilus* is mediated because it is not a major probiotic molecule in Euglena [117]. This data may be important in the development of fermented milk drink formulations with β-glucan from microalgae *Euglena gracilis*.

The effectiveness of bacterial β-glucan was evaluated in yogurt by Niamh Kearney et al. [118], who used the strain *Lactobacillus paracasei NFBC 338* containing the *Pediococcus parvulus* glycosyltransferase gene responsible for β-glucan production. This technology makes it possible to reduce the syneresis of the fermented clot due to the high moisture-binding capacity of β-glucan and to improve the texture of yogurt due to the increase in viscosity. However, the influence of β-glucan on the vital activity of *Streptococcus thermophilus* and *Lactobacillus delbrueckii ssp. bulgaricus* was not detected, although the recombinant probiotic culture maintained high viability (> 108 CFU mL^−1^) during 28 days of yogurt storage. The improvement in the structure of yogurts when using different lactobacteria as a source of β-glucan can be explained by their production of exopolysaccharides, in particular β-glucan capable of inhibiting casein aggregation, which increases its stability and affects the viscosity of the final product [119].

## 4. The Use of β-Glucan in Cheese and Cheese-like Products Technology

### 4.1. Oat and Barley β-Glucan

β-glucan use of various origins in cheese technology is not as popular as in dairy beverages, which is due to the specific properties of β-glucan in these food systems. Scientists are becoming more interested in it, though, because it can structure mixtures during the fermentation of milk curd and actively bind whey, which leads to a higher yield of the finished product [108,120].

The most studied in the technology of cheese and cheese-like product manufacturing is cereal β-glucan, which, despite its ability to imitate the taste of milk fat, has certain limitations due to the deterioration of sensory indicators of protein-containing products [121]. Thus, Pantelis Volikakis et al. [77] investigated the effect of oat β-glucan in amounts of 0.7 and 1.4% in the technology of white-brined cheese with a reduced fat content (70% lower than in the full-fat analog) on quality indicators. The ability of β-glucan to reduce active acidity and increase the yield of the finished product by preventing whey separation during 90 days of storage was noted, which undoubtedly affects the texture of the product [77]. However, its use leads to a significant deterioration in taste and appearance. Other studies have also reported the possibility of an aftertaste of oat flour and a gray color, which significantly impairs the consumer properties of the product. Thus, the use of cereal β-glucan in cheese technology should be limited and should be combined with natural dyes and/or food flavor fillers to make a product with an interesting and unique taste and smell [122]. The fat replacer "Nutrim" based on oat β-glucan in the form of a hydrocolloid suspension was studied in samples of low-fat cheddar cheese (mass fraction of fat: 3.47 and 6.84%) [123]. Although the control sample had a higher yield of finished product compared to the experimental samples, an improvement in cheese texture was noted. Using scanning microscopy, it has been proven that a β-glucan-based milk fat replacer contributes to the formation of a uniform texture with small moisture droplets, which is associated with the high fat- and water-holding capacity of the polysaccharide presented in the form of a suspension [124,125]. 

The combination of β-glucan and phytosterols makes it possible to obtain an effective replacer for milk fat in low-fat cream cheese technology [126]. β-glucan provides a significant increase in viscosity, while phytosterols reduce the coefficient of friction, which contributes to the easy and plastic spreading of the cheese. This combination makes it possible to effectively use the advantages of both components and obtain a low-fat product with sensory properties similar to a high-fat counterpart. The combination of phytosterols, β-glucan, and the probiotic culture of *L. Rhamnosus* allowed for an increase in the content of diacetyl compounds, which contribute to the formation of the buttery taste of the low-fat cream swirl [127]. In addition, the combination of β-glucan and phytosterols provides an open product structure with evenly distributed cell walls of *L. Rhamnosus* in a casein matrix [127].

That is why the search for options for combining polysaccharides with other technological ingredients has extraordinary practical value, especially in the technology of low-fat products [128,129].

Barley β-glucan has been investigated in the composition of functional low-fat Dahi cheese based on buffalo milk [79]. The mass fraction of the additive at a level of 0.5% ensures the most harmonious combination of individual organoleptic indicators. Adding more barley β-glucan makes the product less similar to traditional cheese because it makes the structure too hard. This happens when β-glucan binds too much whey [130], and it also changes the color of the cheese, which has been seen in other studies [74,120]. R. Elsanhoty et al. [80] note the possibility of using up to 5% of barley β-glucan in low-fat labneh cheese technology, which effectively masks fat reduction (up to 50%) and significantly improves the viability of probiotic cultures *Lactobacillus acidophilus* LA-5 and *Bifidobacteria lactis* Bb12 included in the composition of the starter preparation. An interesting detail of the obtained data is the excellent microbiological indicators, despite the increased yield of the finished product due to the retention of whey [131]. Other scientists have reported on the ability of barley β-glucan to influence the fermentation time of milk cheese mixtures because, at a high degree of gelation between the polysaccharide and the casein micelles of milk, it is significantly reduced [132], which, presumably, can be one of the aspects of enzyme preparation economy and requires additional experimental studies.

The high gelling ability of barley β-glucan has also been investigated in the technology of curd, in particular, due to the urgency of finding natural functional and technological ingredients capable of reducing the volume of raw material losses during production. Carmen M. Tudorica et al. [133] established that barley β-glucan not only reduces the loss of raw materials due to effective water retention [134] but also increases the viscous and elastic characteristics of curd, increases the yield of the finished product, and significantly reduces the duration of the technological process due to the high water-binding capacity and the structuring ability of β-glucan. Furthermore, unlike oat β-glucan, it acts like milk fat, which gives the product a better taste and makes it possible to use more of it [133]. 

Another method of producing cottage cheese with cereal β-glucan is also known [81]. It was established that, with a dosage of β-glucan in the amount of 0.5%, the product acquires excellent physicochemical indicators, and its production becomes more economically profitable than the analog without β-glucan. This is due to the increase in the nutritional value of cottage cheese and the presence of dietary fiber in it, which makes it a functional product attractive to the consumer. The addition of β-glucan also increases the yield of the product and the content of calcium, phosphorus, and vitamins B_2_ and C in it by preventing the separation of whey, which is also a source of biologically active substances [135]. Another important effect of β-glucan is a concern for the environment, which helps to reduce the number of secondary dairy resources [136,137].

The use of barley β-glucan in the technology of pasta filata cheeses requires small doses, which is associated with a negative effect on the elasticity of the cheese mass in the process of kneading and forming products, as well as melting during the use of the finished product [82]. A rational dose of barley β-glucan (0.2%) was established in the technology of low-fat mozzarella, which ensures the high elasticity of the cheese mass due to an increase in the mass fraction of moisture in the product and masks the lack of fat [82].

### 4.2. β-Glucan from Yeast

Although yeast β-glucan has a strong immunostimulant effect and helps protect the body from infectious and viral diseases, its use in cheese technology is problematic because yeast is an undesirable component of the microflora in such products and can cause a variety of texture defects, such as swelling and the appearance of cracks [138,139]. However, β-glucans are more likely to be used in a line of alternative cheeses and products that taste like cheese that are meant to help vegans, vegetarians, people with high cholesterol, and people who like to try new things in their diets. 

Kerry Group P. L. C. (Ireland) offers for use high-quality β-glucan from baker’s yeast, which can be used in the production of cheeses, fermented milk products, and sports drinks [140]. The advantage of this β-glucan is its high content of vitamin B_12_, which is especially important for vegans and vegetarians because it is the most deficient in people who do not consume food of animal origin [141]. "Hyeast Biotech" (China) offers a highly purified preparation of yeast β-glucan, which has a pronounced immunoprotective effect [142]. Such an additive is widely used in vegan pasty cheeses or other cheese substitutes that have a texture and taste similar to their dairy counterparts without the specific odor of yeast due to the high degree of purity [143]. *Saccharomyces cerevisiae* has a significant potential to produce β-glucan. However, its microstructural mesh can be much smaller than certain bacterial species, which should be considered when choosing a food system [144]. Considering that *Saccharomyces cerevisiae* is popular for use in the production of pizza, cheese casseroles, and pasta due to their cheesy taste, β-glucan isolated from them can have a positive effect on the taste properties of cheeses. The preservation capacity of *Saccharomyces cerevisiae* biomass was also reported, which may be related to the content of protein peptides [145,146]. Such a property can be interesting, for example, in the production of cheese with mold.

### 4.3. β-Glucan from Microorganisms

The isolation of β-glucan is also possible from such microorganisms as *Pediococcus parvulus 2.6, Aspergillus spp., Oenococcus oeni IOEB0205, Xanthomonas campestris, Lactobacillus diolivorans G77, Lasiodiplodia theobromae, Botryosphaeria rhodina,* and *Bacillus natto* [147,148]. 

β-glucan produced from *X. campestris* has the smallest dimensions of the microstructural network, which affects the presence of biologically active substances. It is somewhat higher in β-glucans from *S. cerevisiae* and *B. natto*, and the lowest biological activity in β-glucan is from *A. oryzae*.

*Bacillus subtilis natto* is used in Asian countries in the technology of fermented products, in particular cheese-like products based on legumes [149]. Cyclic β-glucans from *Agrobacterium, Bradyrhizobium,* and *Rhizobium spp.* are considered more soluble and bioavailable, but they have not been studied in cheese technology, which outlines the scope of scientific interest in them.

### 4.4. β-Glucan from Edible Mushrooms

The use of β-glucan from edible mushrooms is limited due to insufficient awareness of their immunomodulating and preventive properties and a lack of scientific data on their use in cheese technology [150]. However, considering the fact that cereal β-glucan is not widely used in the composition of these products, edible mushrooms can be a promising source of β-glucans [151].

In addition, β-glucan extracts from the edible mushroom *Pleurotus ostreatus* in the amount of 0.4% were used in the production of non-fat cheese based on sheep’s milk [78]. In general, the texture of the cheese was improved by the β-glucan use of *Pleurotus ostreatus*, but for this, the duration of ripening must be at least 180 days. The most important advantage of its use is the possibility of reducing the mass fraction of fat by up to 50% in cheese, which does not affect the change in organoleptic indicators. This is partly due to the ability of β-glucan to mimic the taste of milk fat [79] and, on the other hand, to the pasty form of β-glucan obtained from *Pleurotus ostreatus* [78]. β-glucan in a form other than powder may pose some problems, but this technology has a place and needs to be improved because of the demand for high-quality, low-fat functional products that taste like their high-fat counterparts [10]. 

Kondyli et al. [83] continued a series of experiments with β-glucan from *Pleurotus ostreatus* in the technology of a functional pasty cheese-like product based on sheep’s milk. At a mass fraction of 0.4%, β-glucan did not significantly affect the biological value of the finished product, but allowed the improvement of its structure by increasing viscosity [83]. However, due to the high moisture-binding capacity of β-glucan, the pasty cheese-like product contains more moisture than its counterparts, which affects the water activity in this food system and, accordingly, the duration and storage conditions of the finished product. In addition, the product was distinguished by a bright and attractive color. Khorshidian et al. [152] recommended not exceeding the dose of β-glucan in dairy products by more than 1%. However, other scientists proved that the permissible dose of β-glucan in yogurt can be 1.5% [74], which outlines the contradiction between the existing scientific data and requires clarification for each product technology individually [83].

Expanding the possibilities of using β-glucan from edible mushrooms is also promising from the point of view of cost. *Schizophylum commune Fr* and *Auricularia auricula Judae* are the mushrooms with the highest β-glucan content and, at the same time, the cheapest species among them [153]. Furthermore, in terms of how they work, some types of fungi are like baker’s yeast (*Saccharomyces cerevisiae*) [153,154]. Therefore, the use of β-glucan of various origins in the composition of cheeses and cheese-like products should be rational and take into account their advantages in these food systems. First of all, we are talking about improving rheological characteristics, which is especially relevant in low-fat or fat-free products [155], as well as enriching the chemical composition with biologically active substances, giving the product a functional focus [156,157,158]. On the other hand, the use of β-glucan as a milk fat mimetic is somewhat limited, as it impairs the taste and color of some types of cheese, which requires its combination with fat replacers, for example, based on protein ingredients (Simplesse D-100, etc.) [121]. It is also important that some types of β-glucan can be useful in the production of alternative milk cheeses for certain groups of the population, which requires additional experimental studies.

## 5. The Use of β-Glucan in the Technology of Ice Cream and Frozen Desserts

### 5.1. Oat and Barley β-glucan

Traditional ice cream is a high-calorie product with a fairly high content of sugars (up to 15–16% sucrose and 4.2–5.5% lactose) and fat (up to 16%), which limits its use for people who are overweight, lactose-intolerant, diabetic or who follow low-fat diets [159,160]. Considering the fact that this dessert is common in most countries of the world, scientists are developing new types of ice cream with reduced fat, milk sugar, probiotics, protein, and sour milk content [161]. A sharp drop in taste quality in low-fat or non-fat frozen desserts is a big problem that needs a complex solution [162,163]

Scientists have said many times that polysaccharides can act like they do not have any fat, and when they are combined with protein ingredients and treated specially, they become good replacements for milk fat [164].

Oat β-glucan is similar to guar gum in its technological and functional properties [165], which allows it to be used in ice cream recipes not only as a milk fat mimetic but also to partially or completely replace the stabilizer.

Marek Aljewicz et al. [165] investigated the possibility of reducing the mass fraction of fat in classic ice cream from 10 to 2.5% using highly purified oat β-glucan. A dose of β-glucan at the level of 1% provides a product that is maximally close to the control sample with high-fat content in terms of sensory indicators [165]. Oat β-glucan increases the overrun and viscosity of ice cream mixes. However, in the case of excessive structuring, the aeration of mixtures with air during freezing may deteriorate, which will reduce overrun and increase the hardness of ice cream. Due to its high moisture-binding and water- and fat-holding capacity, β-glucan in excess effectively structures mixtures, which impairs the uniform distribution of the air phase in the thickness of the product [4,166]. In order to increase the aeration of mixtures during freezing, it is advisable to use inulin, which can reduce the hardness of ice cream, which is one of the recommendations for the use of β-glucan in frozen desserts [167,168].

Lazaridou et al. [169] also dealt with the issue of reducing the effect of β-glucan hardness in food systems. It was established that the addition of polyols to barley β-glucan solutions slows cryostructuring and leads to the formation of weaker and less thermostable cryogels, compared to control systems without polyols. On the other hand, mechanical deformation tests revealed an increase in the hardness and strength of β-glucan cryogels with the inclusion of polyols in the following order: sucrose, fructose < glucose, xylose < sorbitol, which requires further scientific research in the technology of frozen desserts.

The use of cereal β-glucan in the amount of less than 0.5% in ice cream technology may not be justified in general because such a dose will not make it possible to achieve a technological effect. It is well known that using 0.4% barley β-glucan in the production of ice cream based on buffalo milk with 4.17% fat not only produces the desired result but also reduces overall quality, particularly due to the unsatisfactory texture of the product [170,171]. A similar conclusion was reached by another group of scientists, who determined the dose of oat β-glucan at the level of 0.6% to be the most acceptable among the range of 0.1–0.6% for use in low-fat ice cream, which provides ice cream with a rich milky taste due to increased viscosity, which prevents the defect of a watery and empty taste [85].

Rahil Rezaei et al. [87] reported that oat β-glucan was able to regulate the textural properties of frozen soy yogurt by increasing viscosity. In addition, an overall increase in quality was observed when the fermentation process of the mixture was prolonged since the presence of soybeans is an inhibitory factor for the duration of fermentation of such mixtures [172]. With higher concentrations of oat β-glucan in the technology of frozen soy yogurt, the duration and temperature of ice cream mixture ripening are subject to the refinement of technological modes, which will significantly affect the quality of the product. The introduction of β-glucan in the amount of up to 1–2% allows reducing the duration of ripening from 24 to 13 h (at a temperature of 2 °C), which ensures the high viscosity and moderate hardness of the product after freezing [87]. An increase in temperature up to 6 °C does not make it possible to reach the optimal viscosity value within 24 hours and negatively affects the quality of the frozen dessert.

**Figure 4:**
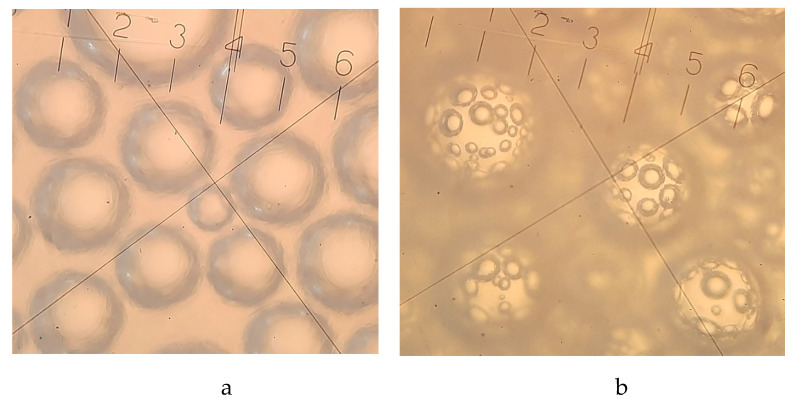
Microstructure of milk–vegetable ice cream: **a**-without β-glucan, **b**-with 1% oat β-glucan [12].

Ice cream is a complex dispersed system in which the air phase is distributed within fairly stable air bubbles in a partially frozen dispersion medium, which influences the formation of ice cream quality characteristics such as overrun, resistance to melting, and texture [173]. Because of this, the authors think it’s important to study in detail the microstructure of ice cream made with oat β-glucan (Figure 4). This is a scientific first and can help experts decide where to go next with research on different kinds of ice cream made with β-glucan. Scientists found that a dose of β-glucan at the level of 0.75–1% contributes to the structuring of low-fat milk–vegetable ice cream mixtures, which is explained by an increase in low-energy bonds between functional groups of polysaccharide macromolecules [12]. The greater number of areas of consecutive cellotriose units that "cross-link" β-glucan macromolecules into the gel matrix [60] increases the time of ultimate failure and, as a result, causes the formation of a plastic ice cream texture. The microstructure of milk–vegetable ice cream is characterized by the presence of an additional framework of microbubbles, which wraps larger air inclusions and stabilizes them (Figure 4). Other scientists [174] also looked at β-glucan’s ability to make foam, but this work [12] is the first to describe how this polysaccharide can make foam with a complex structure.

In another work, it was indicated that, with the help of microscopy, it is possible to determine the limited dose of oat β-glucan in the recipe composition of ice cream [86]. Thus, in the composition of low-fat sour milk ice cream, the optimal amount of oat β-glucan is 0.75%, which ensures an even distribution of the air phase inside the ice cream, a creamy consistency, and increased overrun. However, increasing the dosage of β-glucan to 1% leads to an excessive increase in the viscosity of the ice cream mixture and the corresponding complication of its air saturation during freezing, which is confirmed by the results of microscopy, which indicates the rapid destruction of the junctions of air bubbles.

### 5.2. β-Glucan of Bacterial Origin

As in the case of cereal β-glucan use, β-glucan of bacterial origin also leads to an increase in the resistance of ice cream to melting, which is probably related to the formation of a stable polysaccharide matrix, inside which molecules retain free moisture. However, Marek Aljewicz et al. [84] reported that a dose of β-glucan isolated from *Agrobacterium sp.* at a level of 1% provides the same resistance to melting value as 0.5% highly purified oat β-glucan, which suggests a less pronounced ability of bacterial β-glucan to retain free moisture. This can be a technological advantage of bacterial β-glucan because the resulting ice cream will be less hard than when using cereal β-glucan. For a significant decrease in the mass fraction of fat in ice cream, β-glucan from *Agrobacterium sp.* cannot completely mask its absence, which may somewhat limit its use.

The source of β-glucan can be bacteria such as *Alcaligenes spp., Agrobacterium spp., Paenibacillus spp., Rhizobium spp., Saccharomyces cerevisiae, Candida spp.*, fungi such as *Aureobasidium pullulan*, and *Poria cocos*. However, β-glucan from bacteria and fungi has not yet been explored in ice cream production. Scientists should look into whether or not they could be used to make frozen desserts because their chemical makeup includes biologically active substances and complexes that can reveal protective functions in the human body. Triveni P. Shukla and Gregory J. Halpern [175] proposed a way to reduce the mass fraction of fat in ice cream by replacing it with an emulsified liquid shortening composition containing a gel of dietary fiber, water, and lipid, as well as additional bioactive components, including yeast β-glucan [176]. Using yeast β-glucan makes it possible to make low-calorie ice cream that also has health benefits [88]. This type of ice cream will be in high demand among modern consumers. 

Microalgae *Nannochloropsis oculata, Diacronema vlkianum*, and *Porphyridium cruentum* are usually spray-dried or obtained from biomass, and they are used in food technology as stabilizers, hydrocolloids, and dyes. It is known that they contain proteins and carbohydrates, of which from 14–21 to 40% are β-glucans [177,178], which explains the high technological efficiency of powdered supplements in the production of ice cream. Turkish scientists used the powder of microalgae *Nannochloropsis oculata, Porphyridium cruentum*, and *Diacronema vlkianum* in ice cream formulation in an amount from 0.1 to 0.3% [88], which ensured the production of ice cream with an attractive color and an increased content of biologically active substances, in particular, phenolic compounds. The best-tasting ice cream was made with *Porphyridium cruentum* microalgae.

Therefore, in the process of developing recipes for ice cream with β-glucan, it should be taken into account that such additives are aimed at producing a product with original color and taste and giving it a functional status due to enrichment with bioactive compounds [159,179]. To achieve a technological effect, for example, improving rheological characteristics, it is advisable to combine them with other polysaccharides or additives [180].

## 6. Conclusions

1. A review of the scientific literature on the use of polysaccharides, in particular β-glucans of various types, in the dairy industry indicates their prospects and a limited number of scientific studies in this direction, which outlines the scope of scientific interests.

2. The most studied types of dairy and milk-based products, wherein β-glucan is used, are fermented and non-fermented drinks, especially yogurts, as well as low-fat cheeses. Meanwhile, the technology of ice cream, frozen desserts, and cheese products is the least-researched, especially for β-glucans produced from yeast, fungi, and bacteria.

3. The main functions of oat and barley β-glucans are technological (formation of texture, imitation of the taste of milk fat, increase in viscosity of drinks, improvement of rheological characteristics) and biological (reduction of cholesterol level, positive effect on the intestinal tract, etc.), and β-glucans from yeast and edible mushrooms have pronounced biological functions (positive effect on the immune system, etc.). The properties and functions of β-glucan from bacteria are the least-studied.

4. Based on a review of the scientific literature, the chemical structure of β-glucan of various origins is illustrated, and the recommended doses of β-glucan in dairy and milk-based products are systematized. So, for milk drinks, the total dose of β-glucan is in the range of 1.9–3.0%; for fermented milk products 0.1–0.5%; cheeses and cheese-like products: 0.2–1.4%; ice cream and frozen desserts: 0.5–2.0%.

5. The advantages of oat β-glucan are the ability to form a secondary foam structure in ice cream, to prevent the separation of free moisture during the production of fermented milk products and cheeses, and to act as a milk fat mimetic in low-fat products. Exceeding the recommended content ranges, β-glucan can destroy the structure of the product, give it an undesirable aftertaste or uncharacteristic consistency, etc., which indicates the need to comply with the existing recommendations for the use of β-glucan in the composition of certain types of dairy products.

6. The information systematized in the article has scientific and practical significance for scientists and specialists in the field of food technologies for the further innovative development of dairy technologies and dairy products of increased nutritional value.

## Figures and Tables

**Table 1:** β-glucan content with different sources of origin.

Name of the Source	β-Glucan Content, %	References
Cereal crops		
Oat	4.5–5.5	[15,16]
Cultivated barley	4–6	[9,17]
Wild barley	2–4	[9,18]
Rye	1–2.5	[19,20]
Wheat	< 1	[21,22]
Rice	0.4–0.9	[23,24,25]
Sorghum	0.07–0.2	[26]
Transgenic oats	> 6	[27]
Transgenic barley	6–7.8	[28]
Transgenic wheat	0.18–0.89	[29]
Bacteria		
*Agrobacterium sp. ZX09* (Salecan®)	> 90	[30]
*Paenibacillus polymyxa*	1.06	[31]
Lactic acid bacteria	1.9–14.9	[32]
Fungi		
*Saccharomyces cerevisiae*	55–65	[31,33]
*Sparassis crispa*	43.6	[34]
*Gyrophora esculenta*	22.7	[35]
*Pleurotus ostreatus*, *Pleurotus pulmunarius*	0.21–0.53	[36]
*Gyrophora esculenta, Lentinus edodes, Coriolus versicolor, Ganodenna lucidum, Flammulina velutipes*	2.12–19.66	[37,38]
*Ganoderma lucidum*	45.1	[39,40]
*Aspergillus niger mycelium*	50.9	
Microalgae		
*Durvillaea antarctica*	5–33	[41]
*Euglena*	20–70	[42]
*Scenedesmus obtusiusculus* *A 189*	6.4–19.5	[43]
*Durvillaea antarctica (Chamisso) Hariot*	5–33	[41]

**Table 2:** Recommended doses of β-glucan of various origins in fermented and non-fermented drinks and fermented milk products.

Product Name	The Dose of β-Glucan Depending on the Source of Origin			
	Cereal Crops	Yeast	Edible Mushrooms	Microalgae
Chocolate milk (with stabilizer)	3% ^1^ [64]	-	-	-
High-protein drink	1.9% ^2^ [65]	-	-	-
Functional drink	0.5% ^3^ [66]	0.1% ^4^ [67]	-	-
Kefir, yogurt, fermented milk	0.6% ^1^ [68]	-	-	-
Yogurt	0.3% ^1^ [69]	0.3% ^5^ [70]	0.5 ml per 150 ml of yogurt ^7^ [71], 1.0% ^8^ [72]	no more than 150 mg/100 g ^10^ [73]
Fat-free yogurt	-	1.5% ^5^ [74], 0.2–0.5% ^6^ [75]	0.3% ^9^ [76]	no more than 150 mg/100 g ^10^ [73]
Yogurt drink	-	-	-	no more than 150 mg/100 g ^10^ [73]

^1^ Oat β-glucan; ^2^ Oat flour; ^3^ Barley β-glucan; ^4^ β-glucan from baker’s yeast; ^5^ β-glucan from brewer’s yeast; ^6^ β-glucan from wine lees yeast; ^7^ β-glucan from the pleura of Pleurotus ostreatus and lentinan of Lentinus edodes; ^8^ β-glucan from Ganoderma lucidum mushrooms; ^9^ β-glucan from Pleurotus citrinopileatus mushrooms; ^10^ Euglena gracilis.

**Table 3:** Recommended dosages of β-glucan of different origins in cheeses and cheese-like products.

Product Name	The Dose of β-Glucan Depends on the Source of Origin	
	Cereal Crops	Edible Mushrooms
White-brined cheese	0.7 and 1.4% ^1^ [77]	0.4% ^3^ [78]
Low-fat dahi	0.5% ^2^ [79]	-
Low-fat labneh	5.0% ^2^ [80]	-
Curd	0.5% ^1,2^ [81]	-
Low-fat mozzarella	0.2% ^2^ [82]	-
Pasty cheese-like product	-	0.4% ^3^ [83]

^1^ Oat β-glucan; ^2^ Barley β-glucan; ^3^ β-glucan from the edible mushroom Pleurotus ostreatus.

**Table 4:** Recommended doses of β-glucan of various origins in ice cream and frozen desserts.

Product Name	The Dose of β-Glucan Depends on the Source of Origin			
	Cereal Crops	Bacteria	Microalgae	
Ice cream (2.5% of fat)	0.5–1% ^1^ [84]	1% ^2^ [84]	-	
Low-fat milk-vegetable ice cream	0.75–1% ^1^ [12]	-	-	
Low-fat ice cream	0.6% ^1^ [85]	-	-	
Low-fat sour milk ice cream	0.75% ^1^ [86]	-	-	
Frozen soy dessert	1–2% ^1^ [87]	-	-	
Vanilla ice cream	-	-	0.1–0.3% ^3^ [88]	

^1^ Oat β-glucan; ^2^ β-glucan from Agrobacterium sp.; ^3^ Powder from Nannochloropsis oculata, Porphyridium cruentum i Diacronema vlkianum.

## Data Availability

Not applicable.
